# Sensitive Electrochemical and Thermal Detection of Human Noroviruses Using Molecularly Imprinted Polymer Nanoparticles Generated against a Viral Target

**DOI:** 10.1021/acsami.4c01942

**Published:** 2024-09-12

**Authors:** Sarbjeet Kaur, Pankaj Singla, Amy J. Dann, Jake McClements, Mark V. Sullivan, Minji Kim, Sloane Stoufer, James A. Dawson, Robert D. Crapnell, Craig E. Banks, Nicholas W. Turner, Matthew D. Moore, Inderpreet Kaur, Marloes Peeters

**Affiliations:** † School of Engineering, Merz Court, Claremont Road, Newcastle University, Newcastle Upon Tyne, NE1 7RU, United Kingdom; ‡ Department of Chemistry, Centre for Advanced Studies, Guru Nanak Dev University, Amritsar, Punjab 143005, India; § School of Engineering, Engineering A building, East Booth Street, University of Manchester, Manchester, M13 9QS, United Kingdom; ∥ Department of Chemistry, Dainton Building, University of Sheffield, Sheffield, S3 7HF, United Kingdom; ⊥ Department of Food Science, University of Massachusetts, Amherst, Massachusetts 01003, United States; # Chemistry-School of Natural and Environmental Sciences, Newcastle University, Newcastle upon Tyne NE1 7RU, United Kingdom; ∇ Manchester Metropolitan University, Faculty of Science and Engineering, John Dalton Building, Chester Steet, Manchester, M1 5GD, United Kingdom

**Keywords:** molecular imprinted polymer nanoparticles (nanoMIPs), norovirus, electrochemical impedance spectroscopy (EIS), heat transfer method (HTM), biosensors

## Abstract

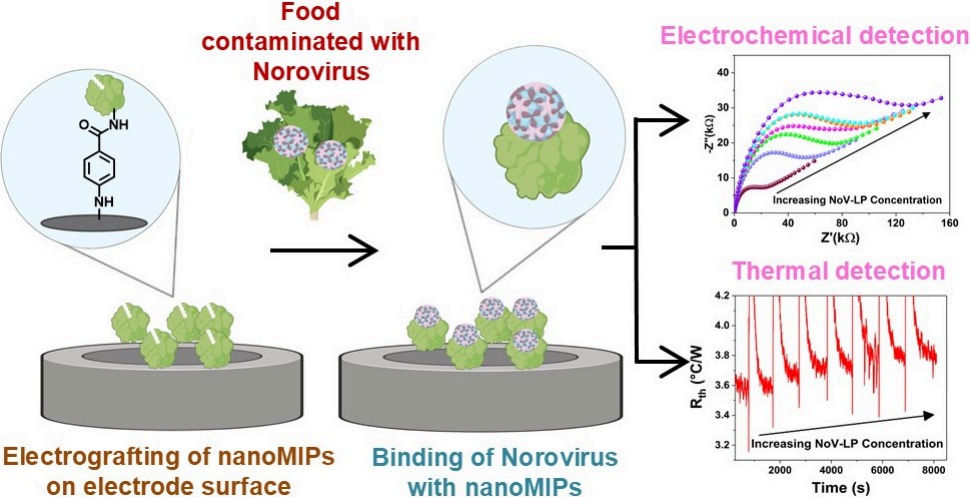

Norovirus (NoV) is the predominant cause of foodborne illness globally; current detection methods are typically expensive, have inadequate sensitivities, and utilize biological receptors with poor stability. Therefore, accurate, cost-effective, and highly stable detection methods are needed to screen for NoV in foods. We developed molecularly imprinted polymer nanoparticles (nanoMIPs) to detect NoV using a small target epitope (12 amino acids) with a solid-phase synthesis approach. The performance of three batches of nanoMIPs with varying monomer compositions (nanoMIP-1, -2, and -3) were compared both experimentally and computationally. Surface plasmon resonance examined nanoMIP binding affinity to norovirus virus-like particles (NoV-LPs), whereby nanoMIP-1 had the lowest K_D_ value of 0.512 μM. This is significant, as traditional targets for generation of norovirus ligands previously reported were generated against drastically larger norovirus capsid segments that have limitations in ease of production. Further, an electrochemical sensor was developed by covalently attaching the nanoMIPs to glassy carbon electrodes. In agreement with our predictions from density functional theory simulations, electrochemical impedance spectroscopy showed a sensitive response toward NoV-LPs for nanoMIP batches tested; however, nanoMIP-1 was optimal, with an excellent detection limit of 3.4 pg/mL (1.9 × 10^5^ particles/mL). Due to its exceptional performance, nanoMIP-1 was immobilized to screen-printed electrodes and utilized within a thermal sensor, where it exhibited a low detection limit of 6.5 pg/mL (3.7 × 10^5^ particles/mL). Crucially, we demonstrated that nanoMIP-1 could detect NoV in real food samples (romaine lettuce) by using electrochemical and thermal sensors. Consequently, the study highlights the exceptional potential of nanoMIPs to replace traditional biological materials (e.g., antibodies) as sensitive, versatile, and highly stable receptors within NoV sensors.

## 1 Introduction

Foodborne diseases (FBDs) are mainly caused by consuming contaminated food or drink, and they pose significant risks to public health worldwide. Millions of people are affected by FBDs each year globally, which results in 420,000 annual deaths and costs of >$110 billion due to medical expenses and lost productivity.^1^ Noroviruses (NoVs) are the most common cause of FBD illnesses worldwide, accounting for approximately 50% of all food-related illnesses.^2^ They are highly contagious and lead to symptoms, such as nausea, vomiting, diarrhea, and stomach cramps. The NoV GII.4 genotype is the most prevalent and has been responsible for 60–80% of NoV outbreaks worldwide in the past two decades.^3,4^ New pandemic GII.4 variants have arisen approximately every few years since the mid-1990s, such as GII.4 Sydney [P31], which has led to numerous outbreaks globally.^5^ A prevalent strain in the United States (US) is the GII.P16GII.4 Sydney strain (referred to as GII.4 2015), which was responsible for over 50% of NoV outbreaks from September 2016 to October 2017.^6^ According to the US Centers for Disease Control and Prevention, between August 1 and October 9, 2023, NoroSTAT-participating states reported 100 NoV outbreaks.^7^ In contrast, during the corresponding period in the previous year, these states reported 71 such outbreaks.

For most people, NoV infection symptoms are self-limiting; however, they can be fatal, particularly in vulnerable populations.^8^ One key method of preventing and managing NoV outbreaks is testing in food and environmental samples.^9^ However, testing can be challenging as food samples present a complex matrix with a wide array of properties and potential inhibitors (e.g., pH, salt levels, fat content), NoV is genetically diverse with numerous genogroups, and the NoV infectious dose is generally very low.^10^ The current gold standard method utilized for NoV detection is the reverse transcription-quantitative polymerase chain reaction (RTqPCR), which generally requires a laboratory environment for analysis with costly apparatus and trained personnel. Furthermore, it can often be slow to produce results as samples must be transported to the laboratory.^11^ Consequently, this significantly limits the availability of NoV testing, particularly in low-resource environments where the health implications of NoVs are most significant.^12^

Due to the distinct issues associated with current laboratory-based NoV testing methods, there has been a major drive to develop portable, accurate, rapid, and cost-effective sensors for point-of-use NoV measurements.^13,14^ These platforms include isothermal amplification methods, such as loop-mediated isothermal amplification (LAMP), nucleic acid sequence-based amplification (NASBA), and recombinase polymerase amplification (RPA), which have demonstrated excellent sensitivity (≥10 copies/reaction) in food and clinical samples with reasonable measurement times (≥30 min) [26–31 min].^15,16^ However, device operation can be complex, and apparatus cost is relatively high, which reduces the potential for widespread adoption, especially in low-resource environments. Biosensors offer simple operation and lower costs compared to isothermal amplification methods; therefore, they are ideally suited for point-of-use measurements. Within the literature, biosensors for NoV utilize a range of detection methods (e.g., electrochemical, optical, magnetic) and receptors (e.g., antibodies, aptamers, peptides).^14,16^ Electrochemical detection offers various advantages, such as excellent sensitivity (≥1 copies/mL), easy operation, and short measurement times (≥10 min).^17−20^ However, using biological receptors in electrochemical biosensors can lead to limited shelf life and stringent storage conditions of the test assays, which hinders their practical use, particularly in hot climates or instances where a cold supply chain is not easily maintained.^21^ Therefore, no emerging point-of-use sensor has gained widespread acceptance as a gold standard for NoV detection.

Molecularly imprinted polymers (MIPs) are synthetic receptors with high-affinity binding sites for a specific target molecule. These binding sites are fabricated by including the target molecule during polymerization. The target is then removed, creating an imprinted cavity with the correct size, shape, and electrostatic surface profile to rebind the target upon exposure.^22,23^ MIPs have numerous benefits as receptors, including binding affinities comparable to or higher than those of antibodies, excellent versatility, and low cost. Consequently, this has enabled their use for the detection of a wide array of targets, such as antibiotics, viruses, and environmental pollutants.^24−26^ A key advantage of MIPs over biological receptors is their excellent environmental stability. For example, MIPs retain their binding affinity even after storage for eight years in ambient conditions. Moreover, their binding affinity is not impacted after exposure to high temperatures (up to 150 °C) or extreme variations in pH and ionic strength.^27^ Therefore, MIPs have significant commercial potential to create sensors without the requirement of limiting storage conditions and an extended shelf life. The properties of MIPs can be further improved by producing MIP nanoparticles (nanoMIPs) using a solid-phase synthesis method, where the target molecule is covalently immobilized on a solid-phase support during polymerization. The advantages of nanoMIPs over conventional MIPs include homogeneous binding sites, fast binding kinetics, and excellent biocompatibility.^28,29^ NanoMIPs have demonstrated exceptional sensing performance for various targets by using different detection methods. However, as nanoMIPs have poor electroactivity, strategies must be employed for their effective use in electrochemical biosensors.^30−32^ For example, an external redox probe may be included in the test solution (e.g., [Fe(CN)_6_]^3–/4–^), conductive nanomaterials may be integrated into the sensors (e.g., carbon nanotubes), or an electroactive probe may be directly incorporated into the nanoMIP matrix during polymerization (e.g., Fe(C_5_H_5_)_2_).^23,32,33^

Human noroviruses are very diverse and contain significant differences in capsid structure that complicate ligand-based detection.^11,34,35^ Although an exhaustive survey of the reactivity of the generated nanoMIPs against a large panel of norovirus genotypes is a valuable and logical next step, the focus of this work is a proof-of-concept to demonstrate the ability of nanoMIPs generated against a short norovirus peptide target to bind not only the peptide against which they were generated but also the P domain of the viral major capsid protein (VP1), and assembled viral capsid, thus multiple targets increase in size/structure. In this study, we present the first nanoMIP-based electrochemical sensor for NoV detection. To comprehensively explore the electroactive capabilities of nanoMIPs, we compared the performance of three nanoMIP types: one standard nonelectroactive batch and two batches with different electroactive probes incorporated into the polymer matrix. The results demonstrated that all three nanoMIP types displayed favorable binding affinity for a range of NoV targets. Furthermore, the optimal nanoMIP exhibited excellent limit of detection (LoD) values of 3.4 pg/mL (1.9 × 10^5^ particles/mL) and 6.5 pg/mL (3.7 × 10^5^ particles/mL) using electrochemical and thermal detection methods, respectively. Consequently, the nanoMIP-based sensor exhibited clear potential for rapid, portable, and sensitive electrochemical detection of the NoVs. Furthermore, the high environmental stability of the nanoMIPs can lead to sensors with an extended shelf life and no limiting storage conditions, which are vitally important for commercialization.

## 2 Experimental Section

### 2.1 Materials

*N*-Isopropylacrylamide (NIPAM), *N*-*tert*-butylacrylamide (TBAM), ferrocenylmethyl methacrylate (FMMA), *N*-(3-Aminopropyl)methacrylamide hydrochloride (NAPMA), *N*,*N*,*N*′,*N*′-tetramethylethylenediamine (TEMED), *N*,*N*′-methylenebis(acrylamide) (BIS), acrylic acid (AAc), (3-aminopropyl)trimethoxysilane (APTMS), Supelco polypropylene solid-phase extraction tubes (60 mL), dialysis cartridges (Vivaspin 20, 3 kDa molecular weight cutoff poly(ethersulfone), potassium hexacyanoferrate(II) trihydrate, potassium ferricyanide(III), and potassium chloride (KCl) were purchased from Sigma-Aldrich (Gillingham, UK). Dopamine methacrylamide (DPMA) was purchased from Polymer Source (Ashwell, UK). Ammoniumpersulfate (APS), Pierce Bicinchoninic assay (BCA) Protein Assay Kit, 1-ethyl-3-(3-(dimethylamino)propyl) carbodiimide (EDC), *N*-hydroxysuccinimide (NHS), succinimidyl iodoacetate (SIA), sodium nitrite, 4-aminobenzoic acid (4-ABA), hydrochloric acid (33%, HCl), phosphate-buffered saline (PBS) tablets, sodium hydroxide (NaOH), acetonitrile, acetone, and methanol were purchased from Fisher Scientific (Loughborough, UK). The epitope sequence CYQESAPAQSDV of the P1 subdomain of the NoV capsid P domain was synthesized and provided by KareBay Biochem (NJ, USA). The P-domain protein of NoV GII.4 Sydney (provided by Dr. M. Moore, University of Massachusetts) was expressed in *E. coli* BL21(DE3) and purified as previously described in Moore et al. with modifications.^34^ Recombinant NoV GII.4 VP1 virus-like particles (NoV-LPs) ab256447 with purity >95% were procured from a commercial supplier Abcam (Cambridge, UK). The NoV-LPs were received in liquid form (100 μg or 0.62 mg/mL) in 0.32% Tris HCl, 0.06% sodium chloride (pH = 7) and for experimental purposes, diluted solutions of NoV-LPs were prepared in PBS buffer (pH = 7). Bacteriophage MS2 (ATCC 15597-B1) and host bacteria *Escherichia coli* (ATCC 15597) were obtained from ATCC, grown and harvested per manufacturer instructions. Similarly, MS2 was quantified with plaque assay per manufacturer instructions. Spheriglass 2429 CP00 glass beads, with a diameter ranging from 53 to 106 μm, were acquired from Blagden Chemicals (Westerham, UK). All chemicals and solvents were of analytical grade and used without purification. Deionized (DI) water (with a resistivity of ≥18.2 MΩ cm) was used for all experiments.

### 2.2 Computational Verification of NanoMIPs

The calculations in this study were completed using density functional theory (DFT) with the Vienna ab initio simulation package (VASP).^36^ A plane wave cut off energy of 520 eV was utilized for the geometry optimization calculations. The projector augmented wave method and the PBE exchange-correlation functional were utilized for all calculations.^37,38^ The Grimme DFT-D3 dispersion correction was also utilized to account for van der Waals interactions.^39^ A *k*-point mesh spacing smaller than 0.05 Å^–1^ was used for the calculations.

Before calculating the binding energies between the monomer and epitope peptide molecules, the structure of each individual molecule was optimized. To reflect the preparation of a nanoMIP, complexes between each functional monomer and epitope were then established. A wide variety of possible binding sites and configurations were considered and simulated for each complex. The magnitude of the binding energy between the molecules in such a complex provides a quantitative indication of the driving force for its formation and stability. The binding energy (Δ*E*_*b*_) was calculated using
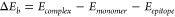
1where *E*_*complex*_ is the energy of a monomer–epitope complex, *E*_*monomer*_ is the energy of a monomer molecule and *E*_*epitope*_ is the energy of the epitope peptide. The more negative the Δ*E*_*b*_ value, the more stable the complex structure and thus the higher the predicted binding affinity.

### 2.3 Synthesis of NanoMIPs

We synthesized nanoMIPs using a solid-phase synthesis approach to target the NoV-GII.4 strain implicated in human disease. The solid-phase method is well established and results in the generation of high-affinity nanoMIPs. Furthermore, it is also a cost-effective technique for medium-scale polymer production due to the reusability of the template. The structure of NoV comprises the major structural protein VP1, which folds into a shell (S) and a protruding (P) domain. The P-domain is further divided into P1 and P2 domains. Among NoV strains, the P2 domain is the least conserved region of VP1 and exhibits high variability in its sequence.^40^ The NoV P1 subdomain is the more conserved region in norovirus capsid P domain and thus would likely enable ligands able to broadly detect a range of norovirus genotypes so that positives for all human noroviruses would occur with the generated ligands.^11,40^ The S domain of the viral capsid has more conserved regions but would not likely be sterically accessible with the generated nanoMIPs. Thus, the design of the nanoMIPs involved a strategic selection process, focusing on choosing an epitope sequence as a template located within the P1 domain.^40^ The selection was made due to the repetition of this sequence across various strains of NoV, which could expand its application in generic detection. However, in this study, we exclusively focused on detecting the GII.4 strain to demonstrate a proof-of-concept nanoMIP-based sensor. To synthesize the nanoMIPs, the surface exposed epitope sequence YQESAPAQSDV (aa 462–472) corresponding to the P1 domain of the NoV GII.4 strain was selected and a cysteine residue was attached at the N-terminus of the sequence to enable immobilization of the epitope peptide on silanized glass beads. This epitope was selected based upon a previous report mapping the interaction site of a broadly reactive monoclonal antibody that displayed cross-reactivity against norovirus GI and GII genogroups, thus allowing the potential for generating nanoMIPs most likely to broadly detect human noroviruses.^40^ The chosen epitope exclusively belongs to the comparatively more conserved NoV P1 domain, which would enable the detection of multiple genotypes of NoV thus enhancing versatility of the assay. It was decided to use a short amino acid fragment (a linear epitope) for the synthesis of the nanoMIPs due to its lower cost and enhanced robustness; longer amino acid fragments possess a specific 3D conformation and are thus susceptible to environmental variations such as changes in pH, temperature, and solvent composition. Linear epitopes have been widely used for nanoMIP synthesis, and a report by Canfarotta et al. used a linear epitope of epidermal growth factor receptor (EGFR) to produce high affinity nanoMIPs for the intended target with excellent thermal and chemical stability.^41^ To the authors’ knowledge, this is among the smallest norovirus capsid protein targets used to generate ligands as primarily the smallest targets reported hitherto are the norovirus P dimer,^42^ P particle,^43^ VP1 (major capsid protein),^44^ and virus-like particles.^45^ The epitope imprinting method provides numerous benefits, including eliminating the high costs involved in protein preparation and purification, potential for future use of target peptide cocktails or various strains to create more broadly reactive ligands, and adaptability to various synthetic conditions such as temperature, pH, and solvents.

**Figure 1:**
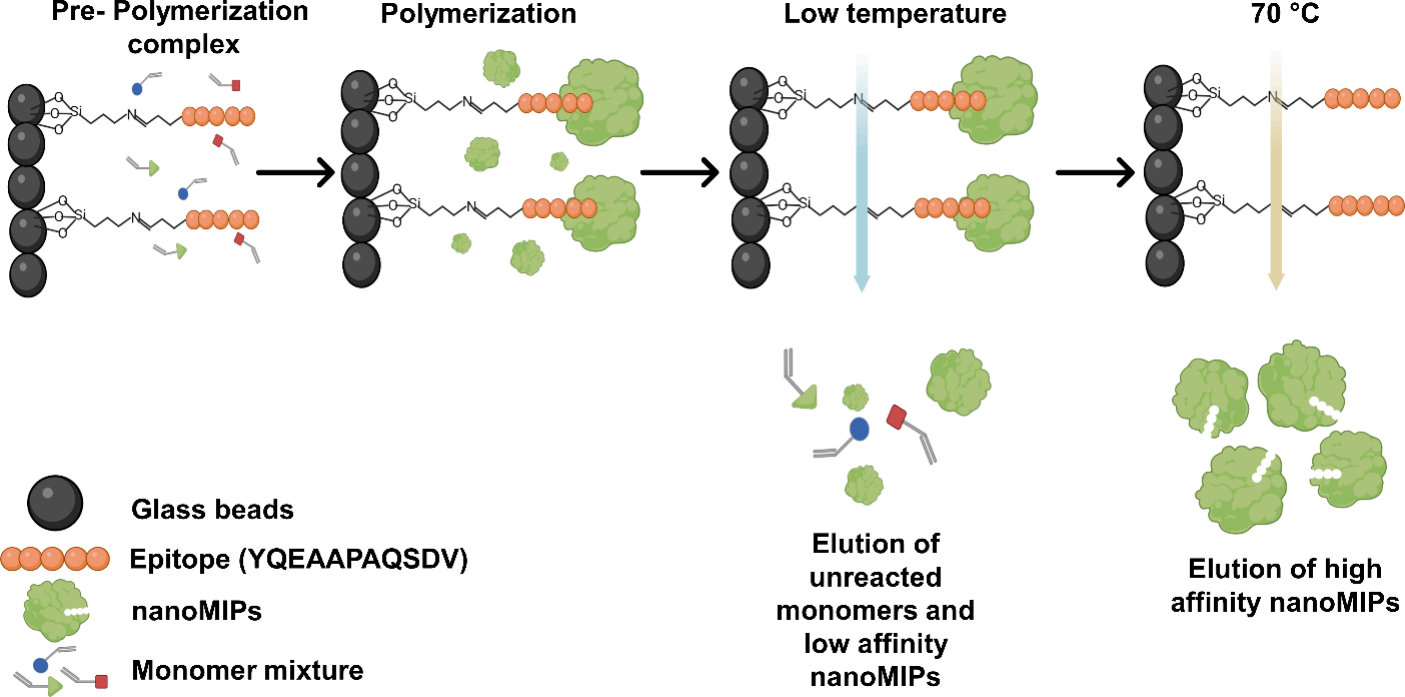
Schematic presentation for synthesis of the three nanoMIP types using the solid-phase approach.

After epitope selection, the nanoMIPs were synthesized using a solid-phase method described in the literature (Figure 1).^28^ This began by activating 60 g of glass beads (solid-phase support) by boiling them in 24 mL of 2 M NaOH for 20 min and then washing them thoroughly with DI water until the washed solution reached pH = 7.4. The glass beads were then washed twice more with DI water and acetone (100 mL each) and dried at 80 °C for 2 h. For silanization, the beads were submerged in 2% (*v*/*v*) APTMS in toluene (24 mL) for 12 h and then washed with acetonitrile (8 × 100 mL) and methanol (2 × 100 mL). Following silanization, the selected epitope was immobilized to the glass beads by initially incubating the beads in an SIA solution (0.2 mg/mL in acetonitrile) for 2 h under dark conditions. The beads were then washed with acetonitrile (400 mL) and incubated overnight in 24 mL of PBS (pH 8.2) containing 10 mg of the epitope and 5 mM of EDTA. Afterward, the epitope-immobilized glass beads were washed with I water (5 × 100 mL) and dried under vacuum. The BCA assay was employed to confirm epitope immobilization on the glass beads, as it is a quantitative method to determine and quantify the amount of peptides present in a particular sample. The peptide bonds present in the epitope reduce Cu^2+^ ions from copper(II) sulfate to Cu^1+^ present in the BCA reagent. The amount of Cu^2+^ reduced is proportional to the amount of peptide in the solution. Upon subjecting epitope modified glass beads to BCA assay, we noticed a purple color change, which indicated that the successful functionalization of the epitope on the solid-phase. The functionalized glass beads were subsequently used for nanoMIP synthesis. The three types of nanoMIPs (nanoMIP-1, nanoMIP-2, and nanoMIP-3) were synthesized by altering the quantities of monomers (Table 1).

**Table 1:** Composition of Functional Monomers Used to Synthesize NanoMIP-1, NanoMIP-2, and NanoMIP-3

monomers	nanoMIP-1	nanoMIP-2	nanoMIP-3
NIPAM	20 mg	20 mg	20 mg
TBAM	17 mg	17 mg	17 mg
NAPMA	4 mg	4 mg	4 mg
FMMA		20 mg	
DPMA			20 mg
AAc	1.1 μL	1.1 μL	1.1 μL
BIS	1.5 mg	1.5 mg	1.5 mg
TEMED	15 μL	15 μL	15 μL
APS	24 mg	24 mg	24 mg

The molecular structures of the monomers used for the different nanoMIP types are shown in Figure S1. A variety of monomers were employed in the synthesis of nanoMIPs, each carefully chosen to elicit specific interactions with the amino acids present in the epitope under investigation. *N*-Isopropylacrylamide (NIPAM) was used for hydrogen bonding, *N*-*tert*-4-butyl acrylamide (TBAM) was used for hydrophobic interactions, and *N*-(3-aminopropyl)methacrylamide hydrochloride (NAPMA) and acrylic acid (AA) were used for ionic interactions. Ferrocenylmethyl methacrylate (FMMA) and Dopamine methacrylamide (DPMA) possesses electroactive properties and was therefore added in the monomer mixture of nanoMIP-2 and nanoMIP-3, respectively, to test their impact on nanoMIPs performance in electrochemical studies. For nanoMIP-1, synthesis began by dissolving 20 mg of NIPAM, 4 mg of NAPMA, 1.5 mg of BIS, 17 mg of TBAM (predissolved in 1 mL ethanol), and 1.1 μL of AAc in 100 mL of PBS (5 mM, pH = 7.4). The same composition was used for nanoMIP-2 and -3, except that 20 mg of conductive monomers (FMMA or DPMA, respectively) was added. The resultant monomer mixture solutions were sonicated under a vacuum for 10 min and then purged with N_2_ for 30 min. Next, 30 g of the epitope-immobilized glass beads were added individually to each solution prepared for nanoMIP-1, -2, and -3. The polymerizations were initiated by adding 400 μL of APS aqueous solution (60 mg/mL) and 15 μL of TEMED to the monomer solutions. The reaction mixture vessels were further flushed with N_2_ and sealed. The mixtures were kept at room temperature for 4 h during polymerization. Afterward, the solutions were poured into solid-phase extraction cartridges with a frit (20 μm porosity) and washed with DI water (9 × 20 mL) at room temperature to remove low-affinity nanoMIPs and unreacted monomers. Then, 20 mL of prewarmed DI water (65 °C) was added to each solid-phase extraction cartridge and placed in a water bath at 65 °C for 15 min. This step was repeated 5 times until approximately 100 mL of a high-affinity nanoMIP solution was collected. The nanoMIP solutions were concentrated in an oven at 60 °C until they were fully dried. The resulting nanoMIPs were purified using a dialysis cartridge (Vivaspin 20, 3 kDa MWCO poly(ether sulfone)) and washed with DI water (5 × 10 mL) using the same dialysis cartridge. Finally, the nanoMIPs were suspended in 50 mL of DI water for further characterization and testing. The yield per production of the nanoMIPs samples was calculated by evaporating the excess solvent by heating.

### 2.4 Evaluating Performance of NanoMIPs against Target Analytes of Different Size

To gain insight into how the detection process is influenced when the target is changed in size and structure, three distinct targets of NoV were selected for the experiments. These targets comprise the specific small epitope against which the nanoMIPs were imprinted, the NoV GII.4 P domain dimer, and norovirus-like particles (NoV-LPs) of the GII.4 strain, which comprise assembled norovirus capsids without genomic material packaged. Therefore, the targets range from a small epitope sequence to intact virus-like particles, enabling us to examine nanoMIP performance when detecting a complex, full-scale structure of NoV.

### 2.5 Characterization of NanoMIPs

The size distribution of the nanoMIPs was characterized by using dynamic light scattering (DLS). Measurements of nanoMIP hydrodynamic diameters (*D*_*h*_) were performed at 25 ± 0.1 °C using a Malvern Zetasizer Nano ZS instrument (Malvern Panalytical, Malvern, UK) with a scattering angle of 173° and laser wavelength of 632.8 nm. Zeta (ζ) potential measurements were performed using the same instrument and disposable folded capillary zeta cells (product code DTS1070, Malvern Panalytical, Malvern, UK). Transmission electron microscopy (TEM) was utilized to determine the nanoMIP morphology. Copper grids were coated with a nanoMIP solution (40 μg/mL) and imaged using a Hitachi HT7800 120 kV TEM machine (Tokyo, Japan), which was equipped with an EMSIS Xarosa camera (Münster, Germany).

### 2.6 Surface Plasmon Resonance Analysis

Surface plasmon resonance (SPR) was performed to measure the binding affinity of the nanoMIPs for the varying NoV targets. The three nanoMIP types were chemically functionalized on the surface of Au chips (Reichert Technologies, NY, USA) using EDC/NHS coupling chemistry. The SPR chips were prefunctionalized with a carboxymethyl dextran hydrogel layer for easy activation through EDC/NHS and optimal deposition.^46,47^ Any unreacted carboxyl groups on the chip surface were deactivated, and any unattached nanoMIPs were removed by using an ethanolamine solution. The binding kinetics of nanoMIPs toward the target epitope and a nontarget epitope sequence (GAQLVLSQTIIQGATPGGGC) were determined in the concentration range of 4–64 nM, while for the P-domain and NoV-LPs, it was assessed in the range of 8–128 ng/mL, using a Reichert 2SPR (Reichert Technologies, NY, USA). A second control channel with no bound MIP was used as a reference. The obtained SPR responses were then fitted into a 1:1 interaction model (Langmuir fit model) utilizing TraceDrawer software, and corresponding equilibrium dissociation constants (*K*_*D*_) were calculated.

### 2.7 Electrochemical Studies

#### 2.7.1 NanoMIP Immobilization on Glassy Carbon Electrodes

NanoMIP-1 and nanoMIP-2 were electrografted on the surface of glassy carbon electrodes (GCEs) with diameters of 2 mm using our previously established protocol for screen-printed electrodes (SPEs).^31,48^ Briefly, a solution of 4-ABA (2 mM) and sodium nitrite (2 mM) was prepared in aqueous HCl (0.5 M) and mixed gently on an orbital shaker for 10 min. The GCEs were immersed in the solution, and cyclic voltammetry was performed from +0.2 V to −0.6 V at a scan rate of 100 mV/s using Ag/AgCl and Pt wire as reference and counter electrodes, respectively. The GCEs were then rinsed with DI water and dried by using N_2_. An EDC (100 mM) and NHS (20 mM) solution was prepared in PBS buffer (pH = 5) and drop-cast (8 μL) onto the GCE surface. After 30 min, the GCEs were rinsed with DI water and dried with N_2_, and then 8 μL of the nanoMIP-1 and nanoMIP2 solutions were drop-cast on their respective surfaces. After 1 h, the nanoMIP-modified GCEs were rinsed with DI water and dried with N_2_. The cross-linking of nanoMIPs on the surface of GCE was confirmed at each step by recording Nyquist plots in 5 mM PBS (pH 7.2) containing 1 mM [Fe(CN)_6_]^3–/4–^ (1:1 mixture) and 0.1 M KCl.

#### 2.7.2 Electrochemical Measurements

All the electrochemical measurements, viz. cyclic voltammetry (CV) and electrochemical impedance spectroscopy (EIS), were performed using a Reference 3000 potentiostat/galvanostat (Gamry Instruments, PA, USA). A three-electrode system consisting of a GCE as the working electrode, a platinum wire as the counter electrode, and Ag/AgCl as the reference electrode (saturated with KCl) was employed for measurements. EIS measurements were conducted across a broad frequency range from 0.01 Hz to 100 kHz, using an amplitude of 10 mV at the open circuit potential. EIS experiments were performed in 5 mM PBS (pH 7.2) containing 1 mM ferricyanide, 1 mM ferrocyanide, and 0.1 M KCl. The data were processed using Gamry Echem Analyst software, and charge transfer resistance (*R*_ct_) values were calculated by fitting equivalent electrical circuit models comprising a combination of elements, including *R*_s_ (solution resistance), *R*_ct_, *C*_dl_ (double layer capacitance), and *W* (Warburg resistance).

To assess the electrochemical sensing capabilities of nanoMIP-1 and nanoMIP-2-modified GCEs, P-domain and NoV-LPs were diluted in a series of concentrations using PBS from 1 pg/mL to 1 μg/mL and 1 pg/mL to 100 ng/mL (5.7 × 10^4^–5.7 × 10^9^ particles/mL), respectively. Then, 10 μL of the NoV target solutions was dropped onto the functionalized GCEs and incubated for 5 min at room temperature to allow the targets to bind with the nanoMIPs. The number of NoV-LPs present in 10 μL of corresponding incubation solutions is 5.7 × 10^2^–5.7 × 10^7^ particles (Table S1). The electrodes were then washed with PBS to remove any unbound targets and used for EIS measurements. The calibration plots were expressed in terms of Δ*R*_ct_ calculated (*R*_ct_ – *R*_ct_^′^), where *R*_ct_ is the charge transfer resistance value after incubation of the nanoMIP-modified GCE in target solutions and *R*_ct_^′^ is the charge resistance value of the nanoMIPmodified GCE in the absence of any target. The LoD for each experiment was calculated by employing the three-sigma technique (3σ/*S*), where σ is the standard deviation of the measurement at zero concentration of analyte and *S* is the slope of the calibration plot.

To mimic real measurements in food, experiments were repeated using romaine lettuce rinse water as a medium. Romaine lettuce, a high-risk food for NoV, was purchased from a local retailer (Marks & Spencer, Newcastle, UK).^49^ The outside leaves were removed and cut into small pieces (10 × 10 mm^2^), and a 50:1 w/w solution was prepared by adding 24.5 g of PBS to 0.5 g of romaine lettuce pieces in a glass vial. The vial contents were thoroughly mixed by using an orbital shaker at 75 rpm for 5 min, a vortex mixer for 30 s, and a sonicator for 480 s. This process was repeated, and the sample was then filtered (10 μm mesh size) before use. For measurements, lettuce rinse water was initially used as a control, which was followed by addition of a NoV-LP-spiked lettuce rinse water solution (100 ng/mL).

### 2.8 Thermal Measurements

Thermal measurements were performed using a bespoke heat-transfer device operated with LabView software, where a proportional-integral-derivate (PID) controller attached to a power resistor (22 Ω) regulated the signal feedback.^50^ The optimized PID settings were fixed for all experiments at *P* = 1, *I* = 15, and *D* = 0. NanoMIP-1 was immobilized to graphite SPEs using the protocol outlined in section 2.7.1. The graphite SPEs were fabricated via screen-printing a graphite ink formulation (Gwent Electronic Materials Ltd., Monmouthshire, UK) onto a polyester substrate, which was then cured at 60 °C for 30 min.^51,52^ Resin measurement cells were fabricated using stereolithography 3D printing (Anycubic Photon, Shenzhen, China). The nanoMIP-modified SPEs were positioned between a heat sink (heated copper block) and a liquid reservoir in the measurement cells. Two thermocouples were inserted into the cell to measure the heat sink (*T*_1_) and liquid reservoir (*T*_2_) temperatures each second. The thermal resistance (*R*_th_), which indicates target binding at the nanoMIP interface, was then obtained through LabView using eq 2, where *P* is the power required to maintain a heat sink temperature of 37.00 ± 0.02 °C:
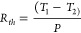
2

The experiments were conducted by simply adding and removing 100 μL of the test liquid from the measurement cell reservoir. PBS was initially added to provide a control *R*_th_ baseline value. This was followed by adding/removing increasingly concentrated NoV-LP- spiked PBS solutions (1 pg/mL-1 μg/mL or 5.7 × 10^4^–5.7 × 10^10^ particles/mL). The corresponding viral particles present in 100 μL of sample is 5.7 × 10^3^–5.7 × 10^9^ particles (Table S1). Each addition was allowed to stabilize for 15 min before its subsequent removal. The thermal experiments produced data plots showing a stepwise increase in *R*_th_ where each plateau represents an increased NoV-LP concentration. These data were presented as dose–response curves by plotting the mean and σ of each stabilized *R*_th_ plateau against NoV-LP concentration, and the LoD value was calculated using the method described in section 2.7.2. Thermal measurements were also performed for detection of NoV in lettuce samples which were prepared using method described in section 2.7.2.

## 3 Results and Discussion

### 3.1 Synthesis and Characterization of NanoMIPs

We have synthesized three batches of nanoMIPs viz. nanoMIP-1, nanoMIP-2 and nanoMIP-3 by varying monomer composition using solid-phase approach. The yield per production of the nanoMIPs was calculated to be 105 ± 0.3, 110 ± 0.4, and 102 ± 0.3 μg/mL, for nanoMIP-1, nanoMIP2 and nanoMIP-3 respectively.

**Figure 2:**
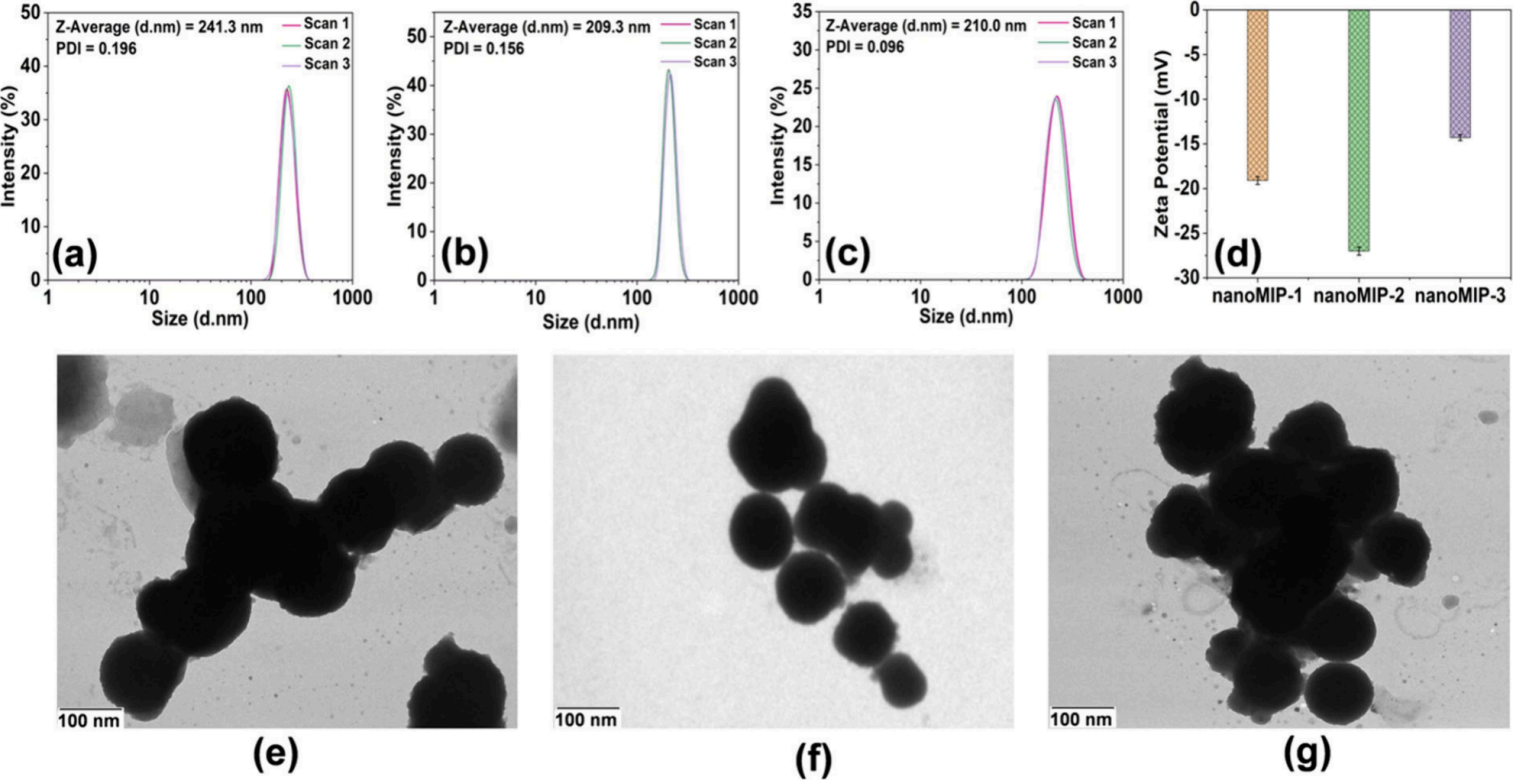
DLS measurements of (a) nanoMIP-1, (b) nanoMIP-2, and (c) nanoMIP-3. (d) ζ Potential of nanoMIP-1, nanoMIP-2, and nanoMIP-3. TEM images of (e) nanoMIP-1, (f) nanoMIP-2, and (g) nanoMIP-3.

DLS reported the *D*_*h*_ of nanoMIP-1, nanoMIP-2, and nanoMIP-3 as 241.3 ± 5, 209.3 ± 7, and 210.0 ± 7.8 nm, respectively (Figures 2(a)–(c)). Furthermore, the polydispersity index (PDI) was 0.196, 0.156, and 0.096 for nanoMIPs-1, nanoMIPs-2, and nanoMIPs-3, respectively. The PDI values were <0.2 for all nanoMIP types, which indicates a high degree of homogeneity among the nanoparticles. The ζ-potential values of nanoMIP-1, nanoMIP-2, and nanoMIP-3 were found to be −19.1 ± 0.43, −27.0 ± 0.45, and −14.3 ± 0.33 mV, respectively (Figure 2(d)), demonstrating that the synthesized nanoMIPs possessed good colloidal stability. TEM measurements showed aggregated particles with spherical morphology for all the nanoMIPs type (Figure 2(e)–(g)), with diameters of 123 ± 8, 101 ± 10, and 117 ± 26 nm for nanoMIP-1, nanoMIP-2, and nanoMIP-3, respectively. This is smaller compared to the size determined by DLS, which is expected since TEM measurements are conducted in the dry state, whereas DLS determines the *D*_*h*_ of swollen nanoMIPs in the liquid. Ultimately, the characterization revealed homogeneous nanoMIPs with excellent colloidal stability and appropriate sizes.

### 3.2 NanoMIP Binding Affinity

**Figure 3:**
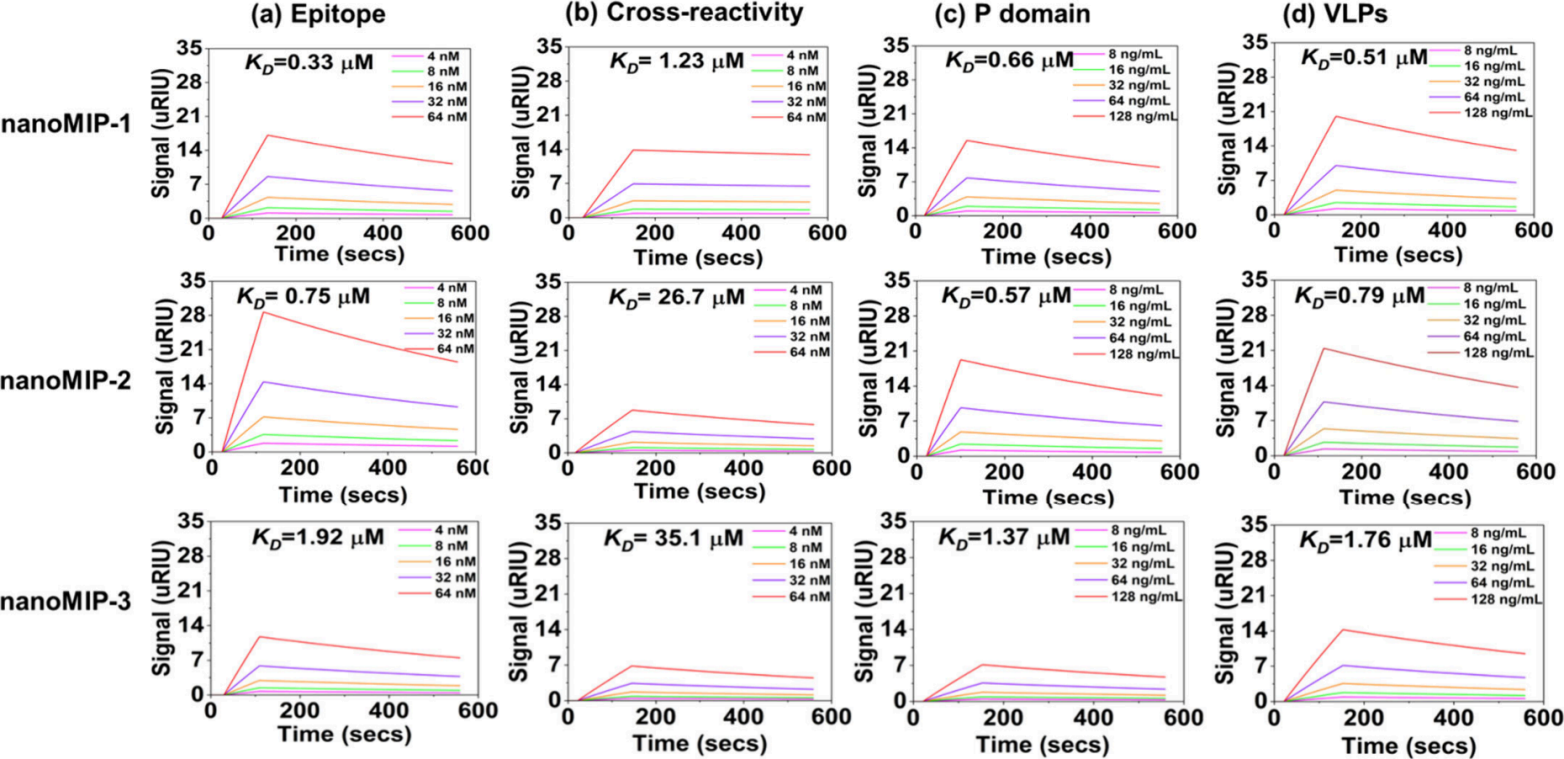
Sensorgrams depicting binding of nanoMIP-1, nanoMIP-2, and nanoMIP-3 with the (a) target epitope, (b) nontarget epitope, (c) NoV P-domain, and (d) NoV-LPs.

SPR was employed to determine the binding affinities of nanoMIP-1, nanoMIP-2, and nanoMIP3 toward the NoV epitope (CYQESAPAQSDV), P-domain, NoV-LPs of NoV-GII.4, and a nontarget peptide sequence (GAQLVLSQTIIQGATPGGGC) with a similar molecular weight to the target epitope. The sensorgrams depicting the interactions between target molecules at five different concentrations and nanoMIPs are shown in Figure 3 and the corresponding *K*_*D*_ values calculated to estimate the binding affinities are tabulated in Table 2. The *K*_*D*_ values for the interaction with the target epitope were calculated to be 0.33, 0.76, and 1.92 μM for nanoMIP-1, nanoMIP-2, and nanoMIP-3, respectively. *K*_*D*_ values are associated with the concentration of nanoMIPs, representing the quantity of nanoMIPs required to interact with the analyte. Consequently, a lower *K*_*D*_ value indicates a greater affinity between the nanoMIPs and the analyte.

**Table 2:** *K*_*D*_ Values for NanoMIP-1, NanoMIP-2, and NanoMIP-3 for a Range of Targets Obtained Using SPR

Sample	Epitope *K*_*D*_ (μM)	Selectivity *K*_*D*_ (μM)	P-domain *K*_*D*_ (μM)	NoV-LPs *K*_*D*_ (μM)
nanoMIP-1	0.328	1.23	0.665	0.512
nanoMIP-2	0.750	26.7	0.575	0.795
nanoMIP-3	1.92	35.1	1.37	1.76

The *K*_*D*_ values for nanoMIP-1 and nanoMIP-2 were comparatively lower than nanoMIP-3, which implies that nanoMIP-1 and nanoMIP-2 exhibit stronger binding affinity toward the target epitope. Selectivity of nanoMIPs plays a pivotal role in diagnostic applications, as it ensures their exclusive binding to the intended target analyte, effectively reducing the occurrence of false positive results. To determine the selectivity of nanoMIP-1, nanoMIP-2, and nanoMIP-3, a nontarget epitope (GAQLVLSQTIIQGATPGGGC) was used, and the results revealed that *K*_*D*_ values reflected a substantial decrease in the affinity. The decrease was 3.7-fold for nanoMIP-1, 35.6-fold for nanoMIP-2, and 18.2-fold for nanoMIP-3 compared to the target epitope, thus demonstrating good selectivity for all three nanoMIP types.

The versatility of the nanoMIPs was also examined by performing binding experiments with the whole NoV P-domain, and the *K*_*D*_ values for nanoMIP-1, nanoMIP-2, and nanoMIP-3 were calculated to be 0.66, 0.57, and 1.37 μM, respectively. These *K*_*D*_ values were comparable to those calculated for the target epitope, which indicates that despite being imprinted against a small epitope of the NoV P-domain, the nanoMIPs exhibited a strong binding affinity for the entire Pdomain. Moreover, for the P-domain, the *K*_*D*_ values for nanoMIP-3 were less than those for nanoMIP-1 and nanoMIP-2, which is the same trend observed for the target epitope. The SPR experiments showed that nanoMIPs exhibited strong binding affinities toward both the target epitope and P-domain of NoV. The subsequent objective was to test the potential of these nanoMIPs toward NoV-LPs of the GII.4 strain, and for this, the experiments were repeated with varying concentrations of NoV-LPs (Figure 3). The corresponding *K*_*D*_ values were 0.51, 0.79, and 1.76 μM for nanoMIP-1, nanoMIP-2, and nanoMIP-3, respectively. Remarkably, these values were comparable to those calculated for the target epitope and P-domain, underscoring the effectiveness of nanoMIPs in detecting the whole virus even when they were imprinted with only a small epitope of the virus’s surface protein.

Moreover, DFT calculations were carried out to verify the performance of the monomers used to synthesize the nanoMIPs by computing their binding energies with the template epitope YQESAPAQSDV. The interactions between NAPMA (present in each nanoMIP), FMMA (present in nanoMIP-2), DPMA (present in nanoMIP-3), and each possible binding site on the epitope were computed to identify which monomer presents the strongest binding energies and to understand the differences in performance between the three nanoMIPs. As shown in Table S2, the strongest binding energies (−78 to −126 kJ/mol) calculated between each monomer and the epitope were found for sites on the serine (S), alanine (A), and valine (V) amino acids. The average binding energies for the most stable monomer-epitope pairs are −84, −70, and −65 kJ/mol for NAPMA, FMMA, and DPMA, respectively. These results showed that FMMA (nanoMIP-2) has better binding affinity with the template epitope than DPMA (nanoMIP-3) and therefore corroborate the SPR findings and observed nanoMIP performance.

These experimental and computational findings have implications for future generation of alternative ligands against noroviruses more generally, as the sorter peptide sequences are able to be synthesized with higher throughput at lower cost; thus, facilitating the ability to generate ligands against cocktails of different norovirus genotypes to potentially generate ligands with broader reactivity. Traditionally, generating ligands that are broadly reactive against the diverse set of human norovirus genotypes has been a traditional challenge.^53−55^ Future work generating other alternative ligands against such a small target in this location would be of value. This outcome highlights the versatility and specificity of nanoMIPs, as they can recognize and bind to the intact virus particles, which are complex and dynamic structures. It means that even a small fragment of a viral protein can serve as the basis for generating nanoMIPs with affinities comparable to those targeting larger regions, thus offering a more cost-effective and resource-efficient approach for virus detection.

### 3.3 Electrochemical Detection

**Figure 4:**
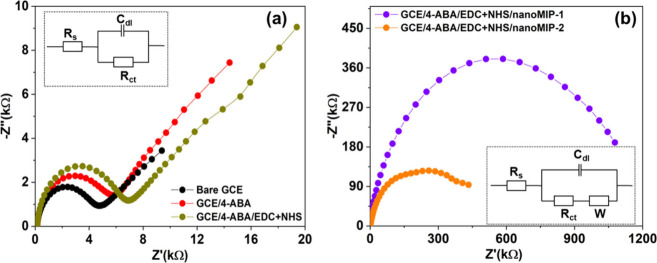
(a) Nyquist plots for bare GCE, 4-ABA electrografted on the GCE (GCE/4-ABA), and activation of carboxylic groups (GCE/4-ABA/EDC+NHS), inset: equivalent circuit. (b) Nyquist plots for covalent coupling of nanoMIP-1 (GCE/4-ABA/EDC+NHS/nanoMIP-1) and nanoMIP-2 (GCE/4-ABA/EDC+NHS/nanoMIP-2), inset: equivalent circuit.

NanoMIP-1 and nanoMIP-2 were selected for electrochemical sensing experiments and thus functionalized to GCEs, as they exhibited superior binding affinities for the NoV targets compared to nanoMIP-3. The covalent coupling of nanoMIPs on the GCE surface was monitored by recording Nyquist plots at each functionalization step (Figure 4(a),(b)), and the corresponding *R*_ct_ values were obtained by fitting circuit models (shown in insets of Figure 4). The *R*_ct_ value is a critical factor for assessing surface conductivity and is graphically depicted as the diameter of a semicircle in the Nyquist plot. A larger diameter means that the surface possesses less conductivity or higher resistivity, indicating a hindrance to the flow of redox probe molecules to the GCE surface. The bare GCE showed a small semicircle at the high-frequency region followed by a straight line at the low-frequency region with a *R*_ct_ value of 4.5 kΩ. This indicates that the electron transfer process is diffusion controlled and there is no substantial hindrance to the flow of redox probe molecules to the GCE surface. Upon electrografting of 4-ABA onto the GCE, the *R*_ct_ value increased to 6.2 kΩ, and a further increase occurred to 7.1 kΩ due to the activation of −COOH using EDC/NHS. When nanoMIP-1 and nanoMIP-2 were immobilized on the GCE, the *R*_ct_ values increased dramatically to 971.0 and 403.5 kΩ, respectively, indicating a significant reduction in surface conductivity.

**Figure 5:**
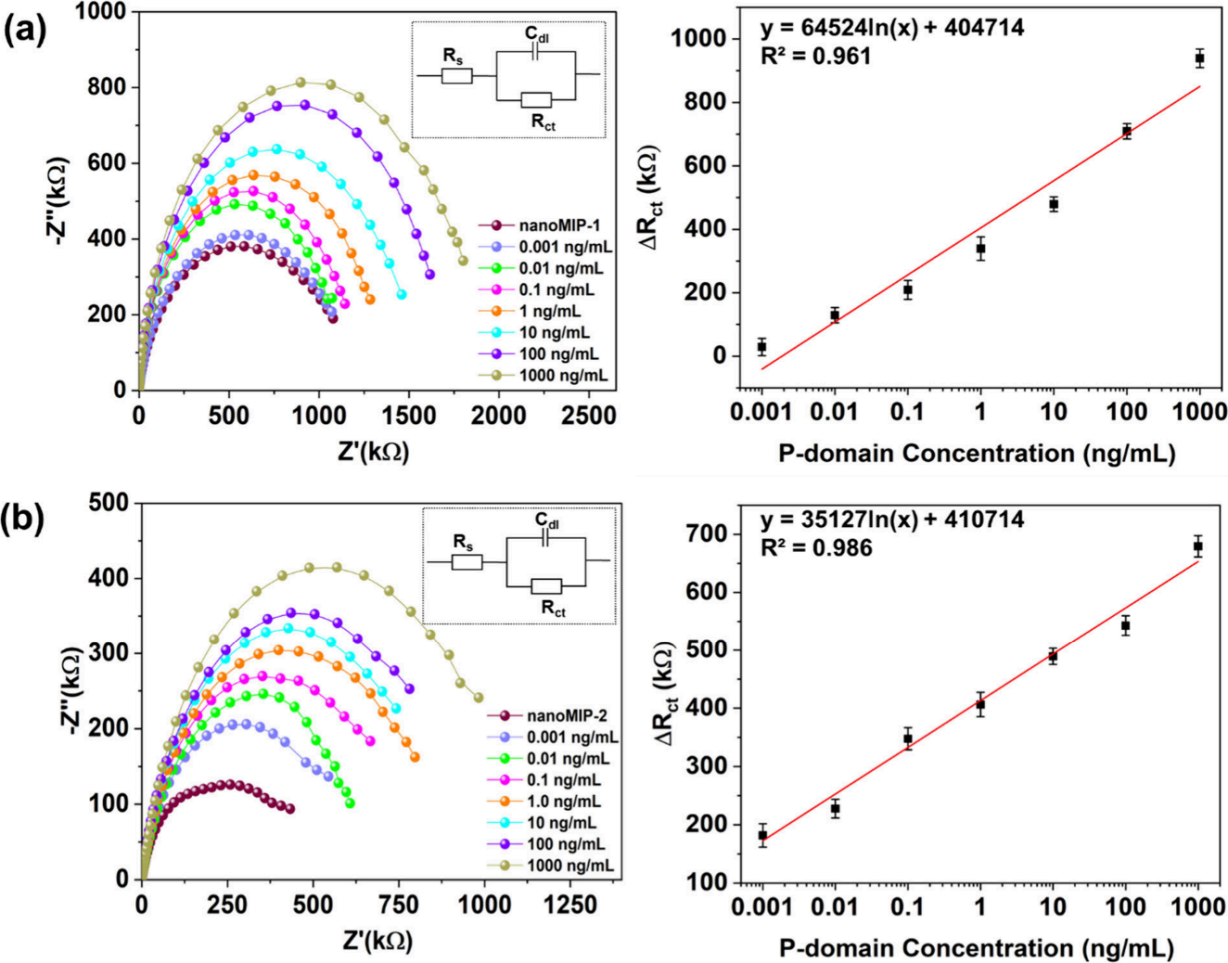
Nyquist plots and corresponding dose–response plots showing the electrochemical detection of P-domain concentrations of 1 pg/mL to 1 μg/mL using (a) GCE/4ABA/EDC+NHS/nanoMIP-1 and (b) GCE/4-ABA/EDC+NHS/nanoMIP-2; insets: equivalent electrochemical circuits. The error bars represent the standard deviation of the triplicate measurements.

After confirmation of their immobilization on the GCEs, nanoMIP-1 and nanoMIP-2 were utilized for electrochemical detection of NoV. The experiments were conducted for detection of P-domain and NoV-LPs and not the target epitope to assess the capability of nanoMIPs to detect larger targets despite being initially imprinted against a smaller epitope. First, the concentration of the P-domain was sequentially varied from 1 pg/mL to 1 μg/mL in PBS, and the corresponding Nyquist plots were recorded (Figure 5). The resulting impedance signal intensities of nanoMIP-1 and nanoMIP-2-modified GCEs progressively increased with increasing concentrations of P-domain. This is because the P-domain attaches to the imprinted sites of the nanoMIP-based sensors, which hinder the transfer of electrons from the redox probe to the GCE surface. The *R*_ct_ values showed good linearity (*R*^2^ = 0.961 and 0.986 for nanoMIP-1 and nanoMIP-2, respectively) over the concentration range and excellent sensitivities (LoD = 1.2 and 5.0 pg/mL for nanoMIP-1 and nanoMIP-2, respectively) were obtained for both the nanoMIPs.

**Figure 6:**
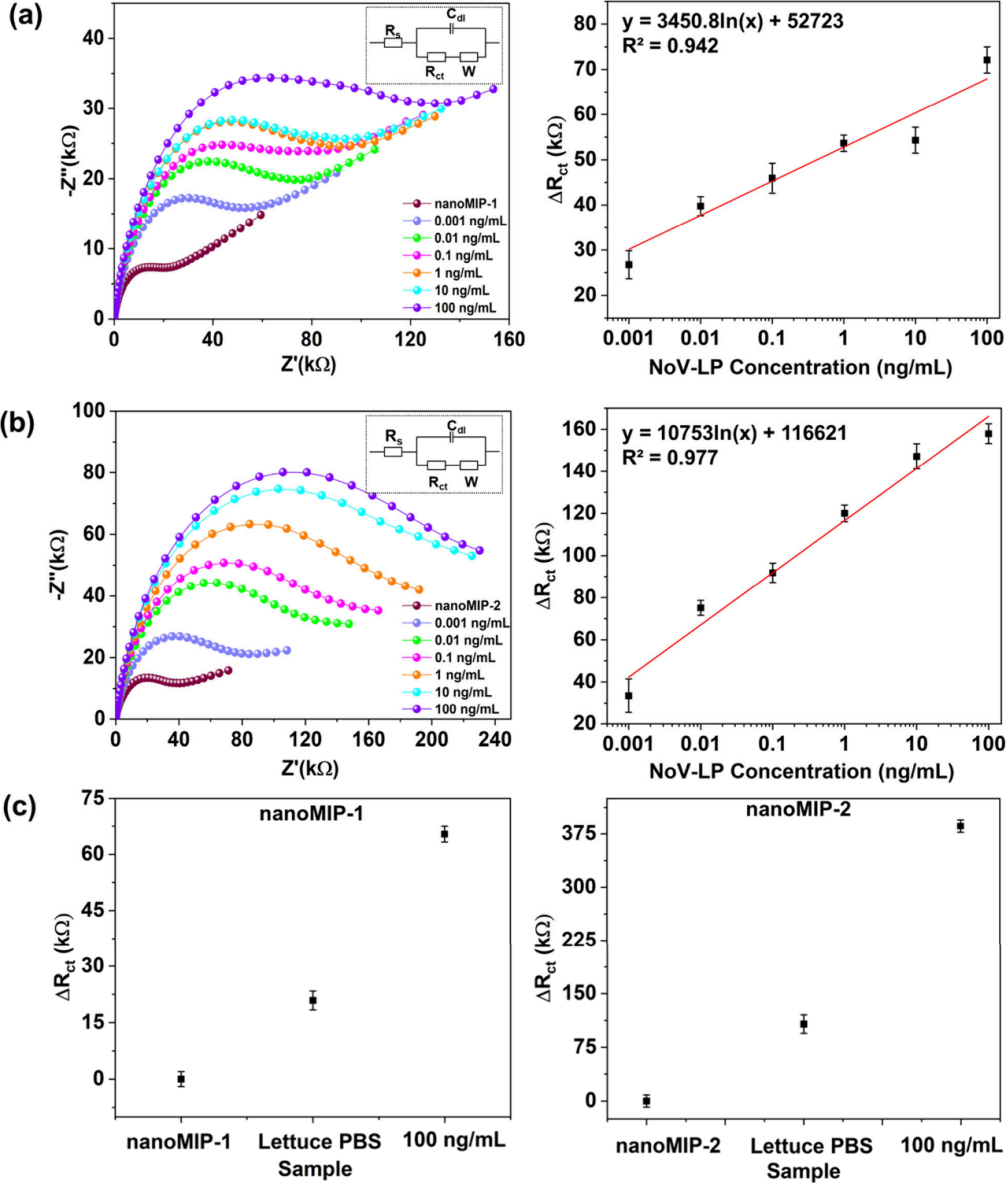
Nyquist plots and corresponding dose–response plots showing the electrochemical detection of NoV-LP concentrations of 1 pg/mL to 100 ng/mL using (a) GCE/4ABA/EDC+NHS/nanoMIP-1 and (b) GCE/4-ABA/EDC+NHS/nanoMIP-2; insets: equivalent electrochemical circuits. (c) Δ*R*_ct_ values for GCE/4-ABA/EDC+NHS/nanoMIP-1 and GCE/4ABA/EDC+NHS/nanoMIP-2 with NoV-LP concentration of 100 ng/mL in romaine lettuce rinsewater. The error bars represent the standard deviation of the three measurements.

The nanoMIP-1 and nanoMIP-2-modified GCEs were also tested for the electrochemical detection of the whole virus using NoV-LPs (1 pg/mL-100 ng/mL or 5.7 × 10^4^–5.7 × 10^9^ particles/mL). The resulting Nyquist plots showed increased *R*_ct_ values with increased NoV-LP concentration for both nanoMIP types due to the progressive occupation of imprinted cavities with NoV, which hindered charge transfer (Figure 6).^56^ A strong linear relationship between Δ*R*_ct_ values and NoV-LP concentrations was observed (*R*^2^ = 0.942 and 0.977 for nanoMIP-1 and nanoMIP-2, respectively) with LoDs of 3.4 pg/mL (1.9 × 10^5^ particles/mL) and 3.9 pg/mL (2.2 × 10^5^ particles/mL) for nanoMIP-1 and nanoMIP-2, respectively.

The nanoMIP-1 and nanoMIP-2 exhibit approximately 4000 times greater sensitivity compared to conventional ELISA assays (LoD ∼ 14.1 ng/mL) for the detection of NoV.^57−59^ We further assessed the sensitivity of our method by comparing it to other techniques reported in the literature (Table S3). Various approaches, such as fluorescent nanostructures,^60^ antibodies,^61−63^ aptamers,^64−66^ optical fibers,^67^ fluorescence quantum dots,^68^ and colorimetric immunoassays using nanohybrids,^58^ have been explored for NoV detection. The majority of sensors listed in Table S3 have LoD values ranging from 10.8 to 80.3 ng/mL. In contrast, the nanoMIP-based sensors in our study boast superior sensitivity with LoD values of 3.4 pg/mL for nanoMIP-1 and 3.9 pg/mL for nanoMIP-2. Comparing our findings with those of Nasrin et al. (LoD of 60 ag/mL) and Lee et al. (LoD of 1.14 pg/mL), their reported sensors are 10,000 and 3 times more sensitive than our current sensor, respectively.^59,69^ However, it is noteworthy that both of their sensors are based on utilization of antibody conjugated on electrode for detection of NoV. Despite the inherent selectivity of antibody or aptamer-based sensors, they have drawbacks such as high cost, labor-intensive preparation, potential for false positives, and issues related to stability and limited shelf life. In contrast, our current sensor technology offers several advantages, including exceptional stability owing to the polymeric nature of nanoMIPs, simple integration procedures, and compatibility with cost-effective mass production methods. Consequently, this demonstrates that our sensor is well-suited for large-scale industrial production and the detection of NoV in real-world applications.

To replicate in-field measurements, we conducted experiments with both nanoMIP-1 and nanoMIP-2 using romaine lettuce rinsewater that had been spiked with NoV-LPs at a concentration of 100 ng/mL (Figure S2). This more complex test was used to confirm that the sensor can effectively function when measurements were performed on real food samples. The results highlight that even in this complex matrix, the sensor displayed an extensive increase in *R*_ct_ values for both nanoMIP-1 and nanoMIP-2 when exposed to the NoV-LP-spiked solution (Figure 6(c)). Also, the sensor exhibited excellent repeatability with only 2.5% and 3.9% standard deviation for detection in romaine lettuce rinsate using nanoMIP-1 and nanoMIP-2, respectively. These observations underscore the sensor’s robustness and ability to perform effectively in real food samples. These results further affirm the sensor’s potential for applications where accurate and reliable detection of NoV-LPs in complex environmental samples, such as food or agricultural runoff, is critical.

### 3.4 Thermal Detection

**Figure 7:**
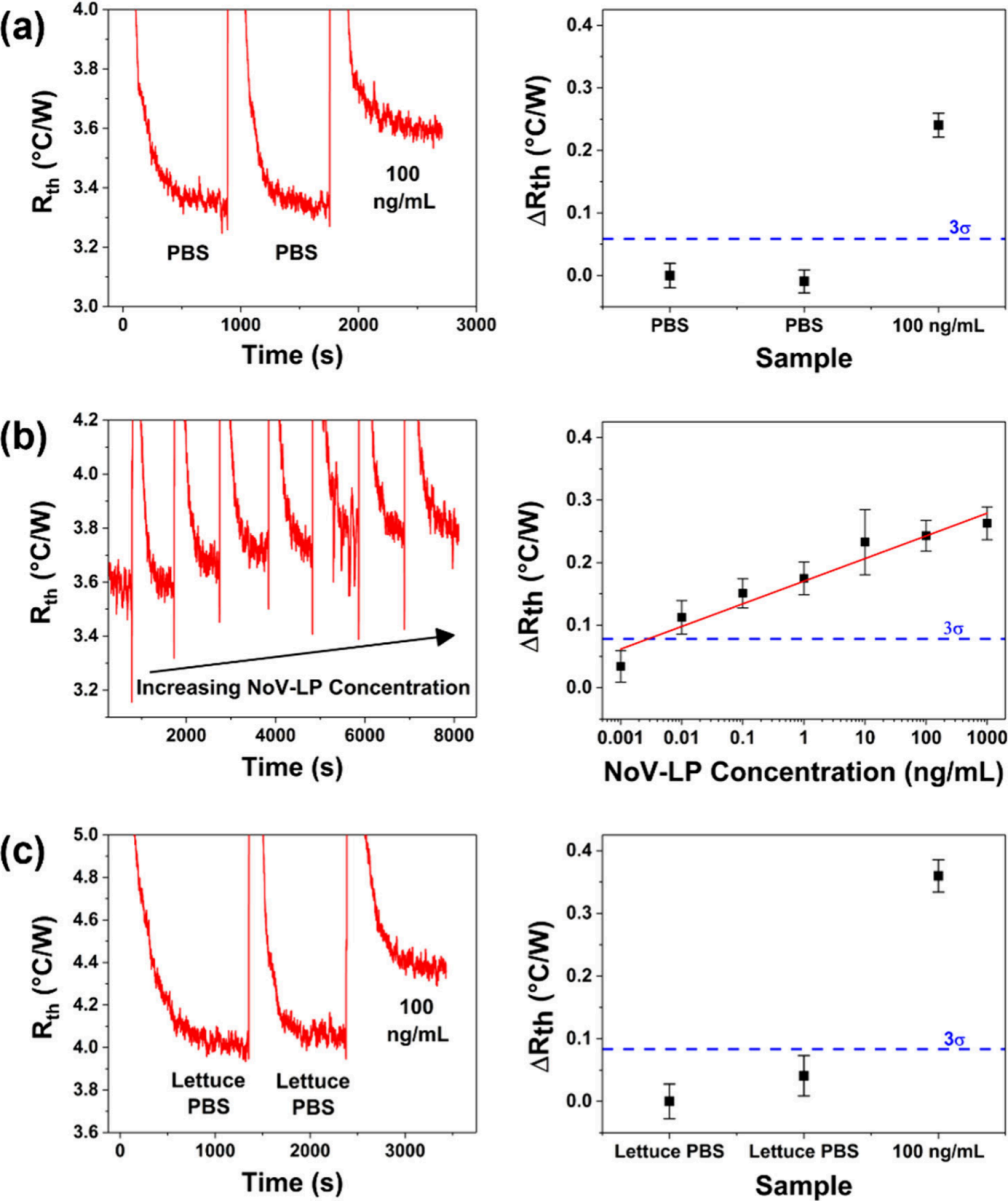
Typical raw data and corresponding dose–response plots show the thermal detection of various NoV-LP solutions using SPEs modified with nanoMIP-1. The 3σ line in the dose response plots represents the baseline σ of the control sample multiplied by three. (a) NoV-LP concentration of 100 ng/mL in PBS. (b) NoV-LP concentrations of 1 pg/mL to 1 μg/mL in PBS. (c) NoV-LP concentration of 100 ng/mL in romaine lettuce rinsewater.

As nanoMIP-1 exhibited superior binding affinity and a lower LoD when tested against NoV-LPs in both SPR and electrochemical experiments, it was also assessed by using thermal detection. This method offers advantages over traditional analysis techniques due to its excellent sensitivity, short measurement times, ease of operation, and low-cost components.^30,48,50^ Furthermore, there is clear potential for integrating thermal detection technology into portable sensors, which would enable point-of-use testing.^70^ Initial thermal measurements revealed a large increase in measured *R*_th_ when NoV-LP-spiked PBS (100 ng/mL) was introduced into the measurement cell, which confirmed significant binding of NoV-LP to the nanoMIP-modified SPE (Figure 7(a)). The full measurement was also completed in less than 45 min, which is far quicker than a conventional ELISA immunoassay (∼several hours).^48^

To comprehensively assess nanoMIP sensitivity, thermal measurements were repeated using a wide range of NoV-LP-spiked PBS solutions (1 pg/mL–1 μg/mL or 5.7 × 10^4^–5.7 × 10^10^ particles/mL). The results demonstrated a clear stepwise increase in *R*_th_ with increasing NoV-LP concentration (Figure 7(b)). Additionally, nanoMIP-1 exhibited excellent sensitivity (LoD = 6.5 pg/mL (3.7 × 10^5^ particles/mL)) that is comparable to many recently developed tests within the literature and ∼1600 times greater than a conventional ELISA immunoassay (LoD = 10.5 ng/mL).^58,59,62^ To evaluate the exclusivity of the best performing nanoMIP sensor (nanoMIP-1), thermal measurements were conducted using the Bacteriophage MS2. The results showed a marginal thermal response when exposed to 10^6^ and 10^7^ particles/mL of Bacteriophage MS2 (Figure S3(a)). However, a significant increase in *R*_th_ was observed with NoV-LPs compared to Bacteriophage MS2, which evidenced the selective binding of NoV-LPs with the imprinting binding sites of nanoMIP-1 (Figure S3(b)). These findings suggested that the developed nanoMIPs sensor exhibited a strong preferential binding/exclusivity to NoV-LPs, highlighting its potential to be used as an efficient molecular recognition element for the development of selective norovirus sensors. To simulate in-field measurements, experiments were repeated using NoV-LP-spiked romaine lettuce rinsewater (100 ng/mL). Despite the more complex test matrix, the sensor still exhibited a significant increase in *R*_th_ for the NoV-LP-spiked solution (Figure 7(c)). Also, for detection in lettuce rinsate, repeated measurements using different electrodes produced only a 5% standard deviation. These results demonstrate the excellent repeatability of the sensor when performing measurements in a real food sample that can vary in composition (such as in lipid composition, ionic strength, etc.). Consequently, the results demonstrated that SPEs functionalized with nanoMIP-1 can be utilized to thermally detect norovirus NoV-LPs with significantly greater sensitivity, repeatability, and shorter measurement times compared to a conventional ELISA immunoassay. Moreover, the sensor maintained its excellent performance when it was tested in real food samples with complex matrices. These thermal results complement the electrochemical detection measurements and highlight the potential of nanoMIPs for in-field measurements.

The results from the electrochemical and thermal measurements are highly promising for using nanoMIPs in norovirus sensor development. However, this is preliminary work and involves only one strain of norovirus (NoV-GII.4). A notable limitation is the absence of inclusivity assays with other norovirus strains. The sensor has the potential to detect other strains of NoV because the synthesis targeted the most conserved region of the P-domain, making it a broadly applicable sensor. This is advantageous given the high extent of contagion of noroviruses and our goal of achieving a simple yes-or-no detection outcome. In future work, we plan to develop new nanoMIP-based sensors for norovirus and conduct inclusivity assays with various NoV strains.

## 4 Conclusions

In this work, we successfully developed a novel nanoMIP-based sensor for NoV that can be used in conjunction with EIS and thermal analysis as a read-out method. As recognition elements, nanoMIPs offer excellent thermal and chemical stability, cost-effectiveness, and superior or equal sensitivity/selectivity compared to conventional biological receptors (e.g., antibodies, oligonucleotides). Three batches of nanoMIPs (nanoMIP-1, nanoMIP-2, and nanoMIP-3) were fabricated using an epitope imprinting approach targeting an amino acid epitope present on the norovirus capsid P1 subdomain. This epitope is one of the smallest targeted for generation of alternative ligands against noroviruses, with advantages over previously reported norovirus target epitopes in reduced cost, higher throughput, and potential for generation of a diverse set of strains’ peptides. The nanoMIPs had *D*_*h*_ values ranging from 200 to 250 nm, and TEM results confirmed their spherical morphology. The *K*_*D*_ values of the nanoMIPs toward the NoV target epitope, whole P-domain, and NoV-LPs ranged from 0.30 to 2.00 μM. The fact binding affinity of ligands generated against such a small norovirus epitope was observed against assembled virus capsid has implications for future generation of ligands against norovirus more broadly. Further, the nanoMIPs with greater binding affinity (nanoMIP-1 and nanoMIP-2) were utilized for electrochemical detection experiments and showed excellent sensitivities toward the P-domain (LoD = 1.2 and 5.0 pg/mL for nanoMIP-1 and -2, respectively), as well as NoV-LPs of the GII.4 strain (LoD = 3.4 pg/mL (1.9 × 10^5^ particles/mL) and 3.9 pg/mL (2.2 × 10^5^ particles/mL) for nanoMIP-1 and -2, respectively). NanoMIP-1 was assessed for its capability to detect NoV-LPs using an innovative and portable thermal method and exhibited a LoD value of 6.5 pg/mL (3.7 × 10^5^ particles/mL), which is lower than a commercial ELISA assay (LoD ∼ 10.5 ng/mL). Ultimately, this technology is highly versatile and can be used to develop biosensors for other foodborne and highly contagious viruses.

## Supporting Information Available

comprising the different functional monomers used in the synthesis, the Nyquist plots for the lettuce rinsate measured with the MIP-based sensors, selectivity studies using thermal detection of Bacteriophage MS2 and NoV-LPs using nanoMIP-1; bar graph showing change in *R*_th_ with of NoV and Bacteriophage MS2-LPs concentrations and additional information about the concentration of NoV-VLP concentration employed, the binding energies between monomers and the epitope calculated using density functional theory, and a comparison of our sensor with current state-of-the-art (PDF)
