# Supplementary material for: Sensitive Electrochemical and Thermal Detection of Human Noroviruses Using Molecularly Imprinted Polymer Nanoparticles Generated against a Viral Target
Source: ACS Appl Mater Interfaces. 2024 Sep 12;16(38):51397–410. doi: 10.1021/acsami.4c01942 (PMC11440458; doi:10.1021/acsami.4c01942)
Supplement: Supplementary file 1 — am4c01942_si_001.pdf [file am4c01942_si_001.pdf]

Supporting Information

Sensitive Electrochemical and Thermal Detection of Human Noroviruses Using Molecularly Imprinted Polymer Nanoparticles Generated against a Viral Target

Sarbjeet Kaur[a,b], Pankaj Singla[a,c], Amy J. Dann[a,c], Jake McClements[a], Mark V. Sullivan[d], Minji Kim[e], Sloane Stoufer[e], James A. Dawson[f], Robert D. Crapnell[g], Craig E. Banks[g], Nicholas W. Turner[d], Matthew D. Moore[e], Inderpreet Kaur[b], Marloes Peeters[a,c]**

[a] School of Engineering, Merz Court, Claremont Road, Newcastle University, Newcastle Upon Tyne, NE1 7RU, United Kingdom

[b] Department of Chemistry, Centre for Advanced Studies, Guru Nanak Dev University, Amritsar, Punjab, 143005, India

[c] School of Engineering, Engineering A building, East Booth Street, University of Manchester, Manchester, M13 9QS, United Kingdom

[d] Department of Chemistry, Dainton Building, University of Sheffield, Sheffield, S3 7HF, United Kingdom

[e] Department of Food Science, University of Massachusetts, Amherst, MA 01003, United States

[f] Chemistry-School of Natural and Environmental Sciences, Newcastle University, Newcastle upon Tyne NE1 7RU, United Kingdom

[g] Manchester Metropolitan University, Faculty of Science and Engineering, John Dalton Building, Chester Steet, Manchester, M1 5GD, United Kingdom

***Corresponding authors:**

Marloes Peeters, Email: marloes.peeters@manchester.ac.uk

Inderpreet Kaur, Email: inderpreet11@yahoo.co.in

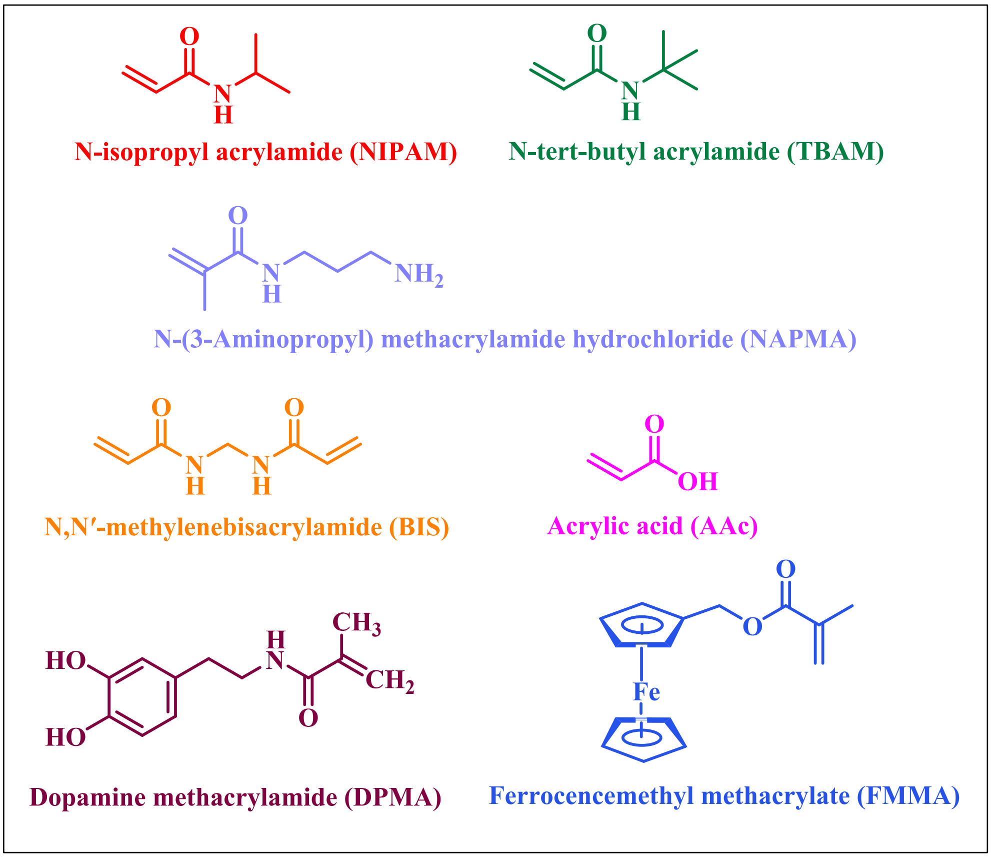

Figure S1. Molecular structures of different functional monomers used for the development of three nanoMIP types.

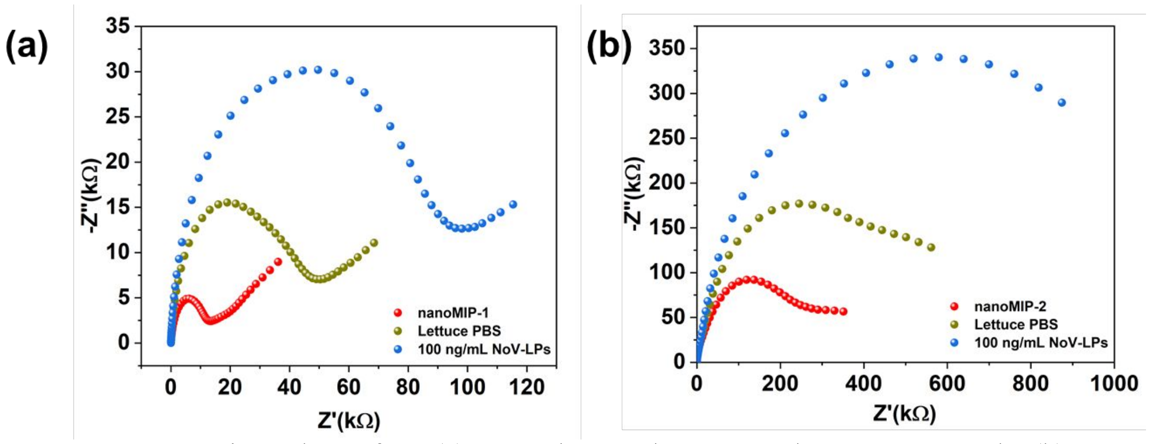

Figure S2: Nyquist plots for (a) GCE/4-ABA/EDC+NHS/nanoMIP-1 and (b) GCE/4-ABA/EDC+NHS/nanoMIP-2 with NoV-LP concentration of 100 ng/mL in romaine lettuce rinse water.

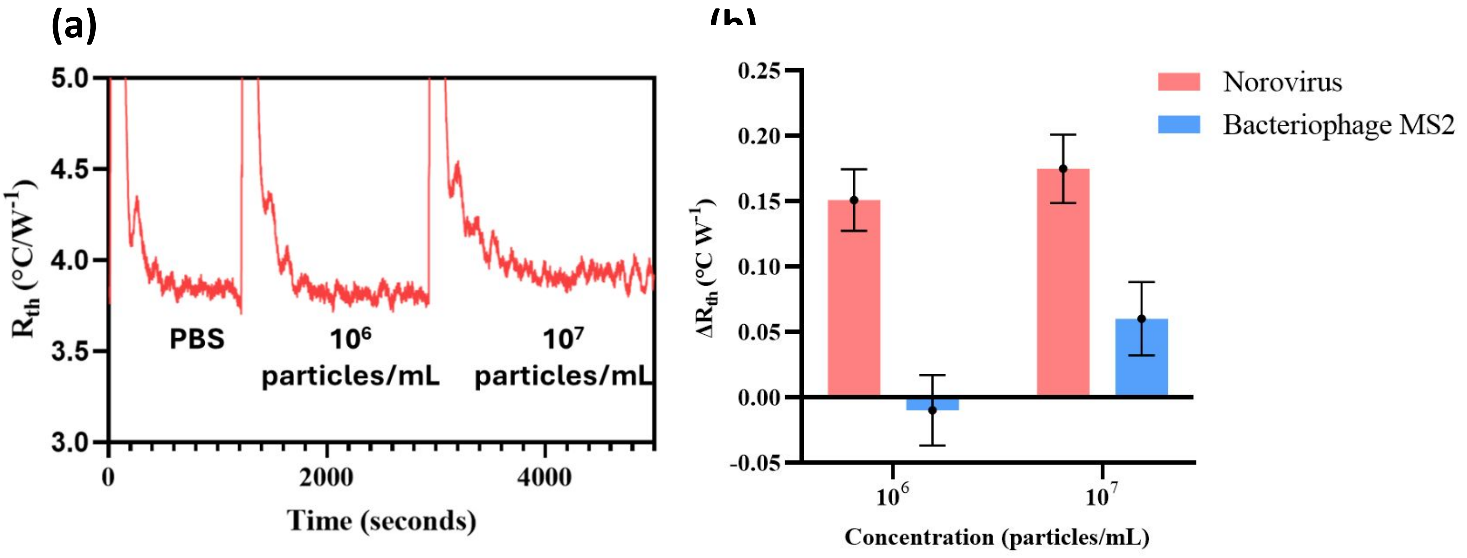

Figure S3: (a) Typical raw data showing the thermal detection of Bacteriophage MS2 (10^6 and 10^7 particles/mL) using SPEs modified with nanoMIP-1; (b) Bar graph showing selectivity in terms of change in R_{th} with NoV and Bacteriophage MS2 concentrations (10^6 and 10^7 particles/mL).

Table S1: Various concentrations of NoV-LPs used for electrochemical and thermal experiments.

NoV-LPs Concentration	Number of NoV-LPs (particles/mL)	Number of NoV-LPs in 10 µL sample for electrochemical experiments	Number of NoV-LPs in 100 µL sample for thermal experiments
1 pg/mL	5.7×10^{4}	5.7×10^{2}	5.7×10^{3}
10 pg/mL	5.7×10^{5}	5.7×10^{3}	5.7×10^{4}
1000 pg/mL	5.7×10^{6}	5.7×10^{4}	5.7×10^{5}
1 ng/mL	5.7×10^{7}	5.7×10^{5}	5.7×10^{6}
10 ng/mL	5.7×10^{8}	5.7×10^{6}	5.7×10^{7}
100 ng/mL	5.7×10^{9}	5.7×10^{7}	5.7×10^{8}
1000 ng/mL	5.7×10^{10}	-	5.7×10^{9}

Table S2: Strongest binding energies (kJ/mol) between monomers and epitope calculated using density functional theory.

Amino acid	NAPMA	FMMA	DPMA
D	−63.68	−41.49	−35.70
E	−71.40	−42.46	−41.49
Y	−65.61	−57.89	−62.72
S	−105.17	−101.32	−100.35
A	−101.31	−116.75	−96.49
P	−66.58	−52.11	−49.21
Q	−70.44	−68.51	−44.39
V	−126.40	−78.16	−88.77

Table S3: Literature reports of NoV detection by various methods.

Method	Technique	Target	LoD	Reference
NoV-LPs-specific antibody-conjugated carbon dots	Fluorescence	NoV-LPs	80.3 pg/mL	60
Monoclonal antibody (mAb)	Photoelectrochemical biosensor	NoV-LPs	0.2 ng/mL	61
GII Ab-immunoassay	Colorimetric	NoV-LPs	10.8 pg/mL	62
S domain protein-specific monoclonal antibody	Electrochemical sensor	NoV-LPs	0.22 ng/ml	63
Modified DNA aptamer	Chemiluminescence	Norovirus GII capsid recombinant	80 ng/mL	64
3D-architectured aptasensor	Electrochemical sensor	NoV-LPs	0.28 ng/mL	65
Norovirus-specific aptamer	Fluorescence	NoV-LPs	4.4 ng/mL and 3.3 ng/mL	66
Optical fibre	Optical transmission	NoV-LPs	1 ng/mL	67
Quantum dot fluorescent dye	SPR-assisted fluorescence sensor	NoV-LPs	0.01 ng/mL	68
Peroxidase-like graphene-gold nanoparticle hybrids	Colorimetric	NoV-LPs	92.7 pg/mL	58
Biotinylated recombinant monoclonal anti-norovirus antibody	Electrochemical sensor	NoV-LPs	60 ag/mL	69
Mouse antiflavivirus group antigen monoclonal antibody	Electrical biosensor	NoV-LPs	1.16 pg/mL	59
